# Fine-grained image classification on bats using VGG16-CBAM: a practical example with 7 horseshoe bats taxa (CHIROPTERA: Rhinolophidae: *Rhinolophus*) from Southern China

**DOI:** 10.1186/s12983-024-00531-5

**Published:** 2024-04-01

**Authors:** Zhong Cao, Kunhui Wang, Jiawei Wen, Chuxian Li, Yi Wu, Xiaoyun Wang, Wenhua Yu

**Affiliations:** 1 School of Electronics and Communication Engineering, Guangzhou University, Guangzhou, 510006 China; 2 School of Life Sciences, Guangzhou University, Guangzhou, 510006 China

**Keywords:** Attention mechanism, Bat, Convolutional block attention module (CBAM), Species identification, VGG16

## Abstract

**Background:**

Rapid identification and classification of bats are critical for practical applications. However, species identification of bats is a typically detrimental and time-consuming manual task that depends on taxonomists and well-trained experts. Deep Convolutional Neural Networks (DCNNs) provide a practical approach for the extraction of the visual features and classification of objects, with potential application for bat classification.

**Results:**

In this study, we investigated the capability of deep learning models to classify 7 horseshoe bat taxa (CHIROPTERA: *Rhinolophus*) from Southern China. We constructed an image dataset of 879 front, oblique, and lateral targeted facial images of live individuals collected during surveys between 2012 and 2021. All images were taken using a standard photograph protocol and setting aimed at enhancing the effectiveness of the DCNNs classification. The results demonstrated that our customized VGG16-CBAM model achieved up to 92.15% classification accuracy with better performance than other mainstream models. Furthermore, the Grad-CAM visualization reveals that the model pays more attention to the taxonomic key regions in the decision-making process, and these regions are often preferred by bat taxonomists for the classification of horseshoe bats, corroborating the validity of our methods.

**Conclusion:**

Our finding will inspire further research on image-based automatic classification of chiropteran species for early detection and potential application in taxonomy.

## Introduction

Horseshoe bats (CHIROPTERA: Rhinolophidae: *Rhinolophus*), comprise over 100 extant insectivorous species with large ears and delicate noseleaves distributed throughout the Old World [1]. They play an important role in ecological functioning, but their taxonomic identification can be challenging due to high inter-species similarity and intra-species variations [1–3]. The unique and characteristic noseleaf structure on their muzzles, with delicate variations in shape and size, has long served as a key feature for taxonomic classification [1, 2]. Recently, horseshoe bats have been found to be natural reservoirs of coronaviruses that can cause highly contagious respiratory diseases in humans, including SARSr-CoV, MERSr-CoV, and 2019-nCoV [4–7]. Horseshoe bats are ranked as one of the potential hosts for monitoring and control of epidemic diseases. However, a significant challenge is species identification for monitoring. Traditionally, chiropteran taxonomists identify species by morphological characteristics, and molecular techniques are utilized to assist in the species identification of highly similar species. The identification of chiropterans thus relies heavily on experts and involves laborious and time-consuming procedures. However, this process has become increasingly challenging due to a shortage of taxonomists specializing in chiropteran classification [8–10].

In the era of big data, image recognition has drawn the attention of many biological disciplines, including species identification, individual recognition, ecological monitoring, and behavior classification [11–18]. This task can be difficult due to the complexities involved in locating, extracting, and distinguishing these features from an image, along with recognizing the unique and distinguishable characteristic features of an object. Fortunately, recent studies have yielded promising results that highlight the potential applications. For example, Peng et al. [19] proposed an object-part attention model for weakly supervised fine-grained image classification (FGIC), which aims to address the challenge of identifying the relationship between positioning and identification. Recently, Li et al. [20] improved the convergence speed and accuracy of the VGG network for vegetable recognition and classification by combining the output features of the first fully connected layers and adding batch normalization (BN) layers. Guo et al. [21] designed a tri-attention network for detecting, identifying, and tracking individuals from videos or still frames of multiple species. This approach achieved an accuracy of 98.70% in facial recognition and 92.01% in individual identification. The challenge other than general image classification lies in localizing and representing the subtle visual differences within various sub-categories, which appear extensively in biological classification. Locating discriminative parts is a key to solving FGIC problems. Attention mechanism can boost the Convolutional Neural Networks’ (CNN) ability to map and recognize fine features of objects. The convolutional block attention module (CBAM) is lightweight and flexible. It can be easily integrated into any CNN architecture and is a simple yet effective choice for improving model performance [22, 23]. However, there is a growing need to overcome the "black box" dilemma in machine learning, particularly in visualizing image information and discriminating regions in the convolution layer of the algorithm. Park et al. [24] utilized the Deep Convolutional Neural Networks (DCNNs) for mosquito classification, achieving up to 97% accuracy. They further enhanced the model’s interpretability by visualizing discriminative regions using Grad-CAM. This approach demonstrated similarity in leveraging morphological features between deep learning models and human experts. These studies mentioned above demonstrate that image classification techniques have promising potential for application in horseshoe bat classification.

In this study, we aimed to explore the effectiveness of the attention mechanism in the VGG16 model for bat species classification. Specifically, we were interested in exploring the ability of the CBAM module to classify 7 common horseshoe bat taxa that exhibit a high degree of interspecific similarity and intraspecific variation. To minimize the computational resources required by the fully-connected layer of VGG16, we modified this layer and adopted global average pooling as the regularization means. This adjustment significantly reduced the number of parameters as well as the computational effort of our network model. In addition, we used the Grad-CAM methods to test whether the visual features captured by the attention mechanism matched the morphological keys used by human experts. Lastly, we compared our network model with other mainstream models for bat classification, analyzing their respective advantages and shortcomings. Our findings provide insights for improving the accuracy of species classification in horseshoe bats.

## Materials and methods

### Image acquisition and training data set processing

Given recent vast variations in the taxonomic system of Chinese bat, we adopt the taxonomic system of Wilson and Mittermeier [1] and Wei [25]. Our image data consisted of photos of living specimens of 7 common *Rhinolophus* bat taxa inhabiting South China. The images were collected between 2012 and 2021 during field surveys and included the following species: *Rhinolophus affinis, R. macrotis, R. nippon, R. pearsonii, R. pernigei, R. pusillus,* and *R. sinicus*. Some easily identified species, such as *R. pernigei*, *R. pearsonii* and *R. pusillus*, these specimens were identified by carefully observing their morphological characteristics, taking photos, and releasing. For the remaining individuals, we first took photos and then released or kept a few specimens to obtain their *CoI*, *Cytb,* or D-loop genes by using molecular techniques to identify them. Permissions for the field surveys in these regions were granted by the related nature reserve administrations and/or the National Forestry and Grassland Administration of China. All samples were captured by following the laws and regulations of China for the implementation of the protection of terrestrial wild animals. Photographs of the specimens including front, oblique, and lateral views were taken using a Nikon D300s/D750/D810 camera with a Micro-Nikkor 105 mm f/2.8G lens under a black background and a built-in flashlight to reduce the influence of environmental light. Each original image for each specimen, a totaling 879 photos, has a resolution of minimum 4288 × 2848 pixels with 24-bit RGB channels.

In order to evaluate the model's performance, approximately one-third of the dataset was randomly selected as the testing set, while the remaining images were used for the training set. To increase the size of the training set and prevent overfitting, we employed image augmentation techniques by using GridMask [26] and Imgagu library including Lambda, GaussianNoise, WithChannels, and GaussianBlur functions. The GridMask was used to augment image diversity by randomly deleting regions of the input images. We then applied the Lambda function to replace every fourth row of the images with black pixels, and the GaussianNoise function to introduce Gaussian noise, adding complexity to the images. Additionally, the WithChannels function was used to rotate the red channel of the image by 45 degrees. Finally, the GaussianBlur function was employed to blur the images using the Gaussian kernel. All images were further normalized to 224 × 224 pixels. These combined techniques were applied during training, which provided a more varied and challenging dataset for our machine-learning model.

### Construction of VGG16-CBAM and Grad-CAM

**Fig. 1:**
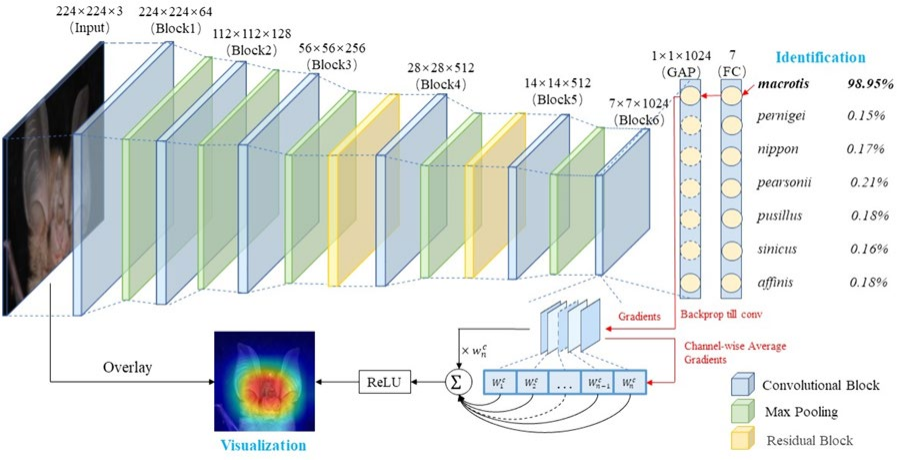
Architecture of the VGG16-CBAM developed for identifying and classifying 7 common *Rhinolophus* species from southern China

Our customized network model is illustrated in Fig. 1. We chose VGG16 [27] with BN [28] as our base model since it has a strong feature extraction capability and has been widely used in image classification tasks. BN was used in VGG16 because it could alleviate the problem of gradient disappearance, act as a regularizer, and speed up the training process of the network, and extraction capability and has been widely used in image classification tasks.

To improve the representation power of VGG16 and the accuracy of our *Rhinolophus* classification task, we added the lightweight attention mechanism CBAM to the VGG16 model. The challenge of increasing the number of layers in deep neural network models, such as VGG16, is the potential emergence of the “vanishing gradient” problem. This issue occurs when the gradients become too small during backpropagation and fail to effectively propagate through the shallow layers, resulting in poor training of those layers. To address this, we incorporated two residual blocks containing CBAM into VGG16 instead of directly attaching CBAM, which enabled a direct path for the backpropagation of the gradient. The residual blocks were inserted after the third and fourth max-pooling layers, and they consisted of convolutional layers and CBAM, which utilized a 7 × 7 convolutional kernel, with 256 and 512 convolutional kernels in the first and second residual blocks, respectively. Furthermore, the convolutional layer before the CBAM could benefit the attention module by aggregating deeper layer information.

Additionally, the fully connected layer of the VGG16 model had many parameters, which consumed significant memory and computational resources. Moreover, an effective method to prevent the gradient from disappearing is lacking [29]. To address these issues, we replaced the fully connected layer with a convolutional layer containing 1024 1 × 1 convolutional kernels, followed by a global average pooling (GAP) [30] operation on the resulting feature map. The output was performed after the fully connected layer consisting of 7 neurons and SoftMax function in turn. By using global average pooling instead of a fully connected layer, we mitigated the risk of overfitting and improved the network's ability to generalize to new data.

**Fig. 2:**
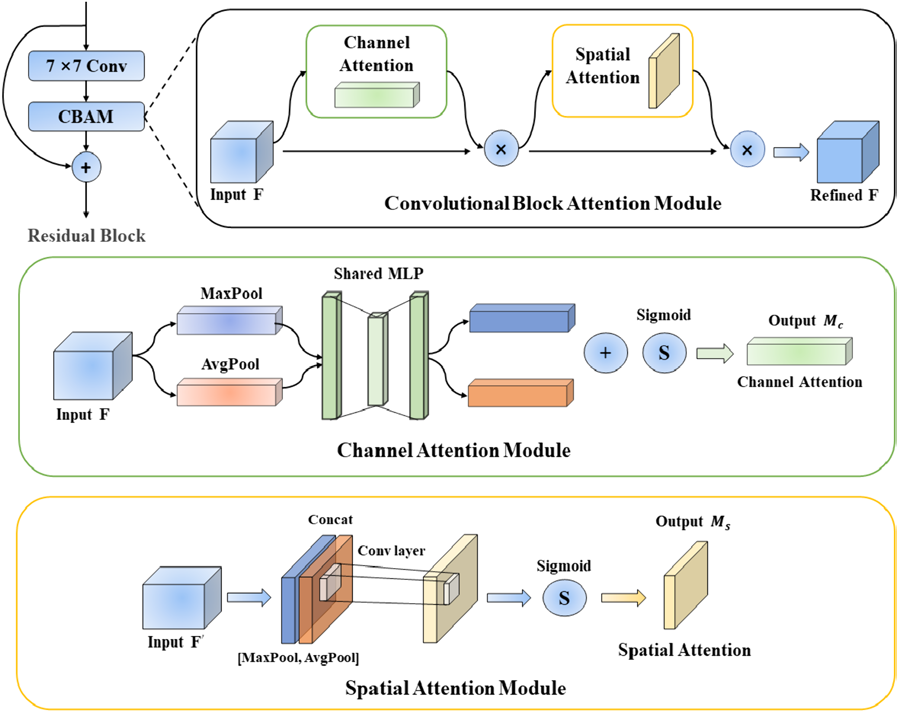
Architecture of the residual block and CBAM developed for identifying and classifying 7 common *Rhinolophus* species from southern China. The residual block consists of a convolutional layer and CBAM. CBAM has two sequential submodules: a channel attention module and a spatial attention module

The CBAM sequentially adopts a channel attention module (CAM) and a spatial attention module (SAM), which emphasizes the meaningful information along the channel and spatial axes (Fig. 2). The CAM squeezes the spatial dimension to aggregate information by max-pooling and average-pooling. Then, it utilizes the results with a shared MLP to produce the channel attention map. Different from CAM, SAM squeezes the channel dimension by two pooling operations and sends the concatenate results to the convolutional layer for a spatial attention map. We further visualized the features of our model that were concentrated via the Grad-CAM [31], allowing us to determine which image regions were important in the classification decision.

### Training model

Model training is a technique for finding the optimal parameters of a machine learning model, such as a neural network. The goal of model training is to minimize the error between the model’s predictions and the true labels of the data, also known as the loss function. The loss function quantifies how well the model fits the data and guides the model update process. In this process, we chose Cross-Entropy as the loss function, which is widely used for classification tasks. Equation (1) shows the formula for the Cross-Entropy loss function.

1 $$\begin{array}{c}{L}_{CE}\left(f\left(x\right),y\right)=\sum\limits_{k=1}^{K} -{y}_{k}\cdot {\text{log}}{f}_{k}\left(x\right)\end{array}$$

where $K$ is the number of classes, ${f}_{k}(x)$ is the probability that the model assigns to the $k$-th class, and ${y}_{k}$ is either 0 or 1 indicating whether the $k$-th class is the correct one.

The optimizer is the algorithm that updates the model’s parameters based on the loss function and the gradients. In training process, we chose the Stochastic Gradient Descent (SGD) algorithm as the optimizer, which is fast and easy to implement and the most widely used optimizer. It updates the model’s parameters by subtracting the learning rate times the gradient, and Eq. (2) shows the formula for SGD. The learning rate was set to 0.01 at the beginning of training, and every 20 epochs the learning rate was updated to the original 0.75.

2 $$\begin{array}{c}{\theta }_{t+1}={\theta }_{t}-\alpha \cdot {\nabla }_{\theta }L\left({\theta }_{t},{x}_{t},{y}_{t}\right)\end{array}$$

where ${\theta }_{t}$ is the model’s parameter at step t, $\alpha$ is the learning rate, $L$ is the loss function, ${x}_{t}$ and ${y}_{t}$ are the input and output of a randomly selected training example, and ${\nabla }_{\theta }$ is the gradient operator to $\theta$.

### Evaluation model

Once the model was trained, we validated the model by feeding the test set into the model for prediction and then calculating the accuracy of the model. Considering the number of images is too small to accurately validate the model using a hold-out approach, we have implemented a K-Fold cross-validation strategy to compare model performance more accurately. K-Fold Cross-validation involves partitioning the dataset into multiple subsets, training the model on different combinations of these subsets, and then averaging these results. This approach allows for a more robust assessment, particularly when dealing with a smaller number of images.

To further evaluate the performance of our proposed model, it was compared against five mainstream convolutional neural networks, including AlexNet [32], MobileNetV2 [33], ResNet50 [34], ViT-B/16 [35]. All models mentioned above used an open-source machine learning framework PyTorch. All were carried out with Python running on an Intel(R) Xeon(R) CPU E5-2620 v4 @ 2.10 GHz and NVIDIA Corporation GV100 [TITAN V] GPU.

Except for Params, Floating Point Operations (FLOPs) and accuracy, we also used F1 score to evaluate these six models. Accuracy is the most intuitive indicator of a model’s classification performance and is defined as the ratio of the number of samples correctly predicted to the total number of samples predicted. Precision is defined as the ratio of true positive samples to the number of all samples that were predicted to be positive. Recall is defined as the ratio of the number of true positive samples to the number of samples actually labeled as positive. F1 score is mainly used to measure both precision and recall, which is defined as the ratio of the product of twice the precision and recall to the sum of precision and recall. Equations (3, 4, 5 and 6) show the formulas for accuracy, precision, recall, and F1 score respectively.

3 $$\begin{array}{c}{\text{Accuracy}}=\frac{{\text{TP}}+{\text{TN}}}{{\text{TP}}+{\text{FP}}+{\text{TN}}+{\text{FN}}}\end{array}$$

4 $$\begin{array}{c}{\text{Precision}}=\frac{{\text{TP}}}{{\text{TP}}+{\text{FP}}}\end{array}$$

5 $$\begin{array}{c}{\text{Recall}}=\frac{{\text{TP}}}{{\text{TP}}+{\text{FN}}}\end{array}$$

6 $$\begin{array}{c}{\text{F1 score}}=\frac{2\;\ast\;\text{Precision}\;\ast\;\;\text{Recall}}{\text{Precision}+\text{Recall}}\end{array}$$

where ${\text{TP}}$ indicates the number of samples predicted to be true positive, ${\text{FP}}$ indicates the number of samples predicted to be false positive, ${\text{TN}}$ indicates the number of samples predicted to be true negative, ${\text{FN}}$ indicates the number of samples predicted to be false negative.

The receiver operating characteristic (ROC) curve, training and validation loss and accuracy curves are other commonly used methods to evaluate the performance of the classification model. The ROC curve plots the true positive rate (TPR) against the false positive rate (FPR) for different classification thresholds. A good classifier has a curve that closely follows the top-left corner of the plot, indicating high TPR and low FPR. The area under the curve (AUC) is a scalar metric that summarizes the overall performance of the ROC curve. A larger area under the ROC curve represents a better classification effect; thus, a larger value of AUC correlates to a better quality of the model. In addition, the training and validation loss and accuracy curves for different CNN models were used to assess their performance during the training process. During model training, we used the cross-entropy loss function, a classical loss function in multi-classification tasks. The loss function estimates the degree of inconsistency between the predicted and true values of the model, and a lower value indicates that the model is learning better. For all models, we plotted these ROC curve, loss function and accuracy by using Matplotlib.

## Results

### The image data set

For 7 *Rhinolophus* species, we collected a total of 879 images (Table 1), with an average of 125 images per species. More than half of the species have at least 100 images, ranging from 56 to 190. For these images, two-thirds of the data set (586 images) was randomly selected as the original testing data, while the remaining images were used for test data. After using image augmentation techniques, the total training data increases fivefold, with an average of approximately 182 images per species. Therefore, our study included a total of 1573 images, with 1280 images used for training models and 293 images used for testing models (Table 1).

**Table 1:** The number of training sets and test sets

	Number of original images	Number of original training sets	Number of images in final training sets	Number of images in test sets
*Rhinolophus affinis*	89	59	145	30
*R. macrotis*	176	117	315	59
*R. nippon*	91	61	141	30
*R. pearsonii*	127	85	186	42
*R. pernigei*	56	36	143	20
*R. pusillus*	190	128	183	62
*R. sinicus*	150	100	167	50
Total	879	586	1280	293

### VGG16-CBAM model performance in 7 *Rhinolophus* species

**Fig. 3:**
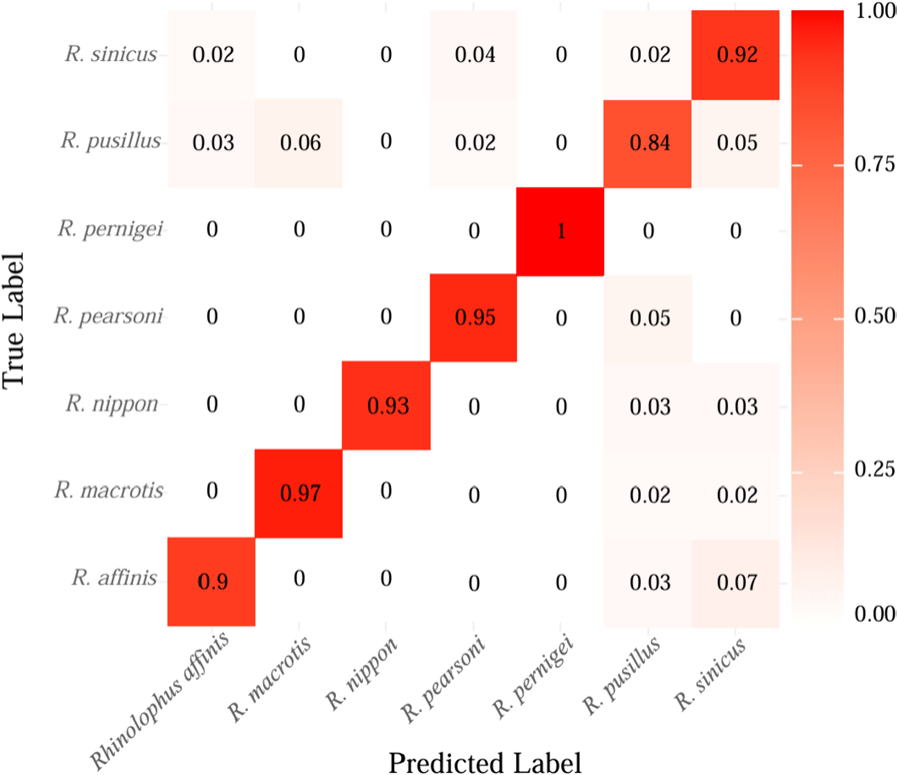
Confusion matrix of the VGG16-CBAM. The horizontal axis is the predicted label, and the vertical axis is the true label

It can be seen that VGG16-CBAM achieved a prediction accuracy of more than 90% for most *Rhinolophus* species, and achieved a promising prediction accuracy of 92.15% from the confusion matrix (Fig. 3). The accuracy of *Rhinolophus pernigei* is 100%, which is highest than other *Rhinolophus* species. However, there were some misidentifications for samples from most species. Samples from *R. pusillus* were the most difficult to classify, with only 84% accuracy. Of those misclassified, 6% were identified as *R. macrotis*, and 5% were identified as *R. sinicus*.

### Comparison of models

**Fig. 4:**
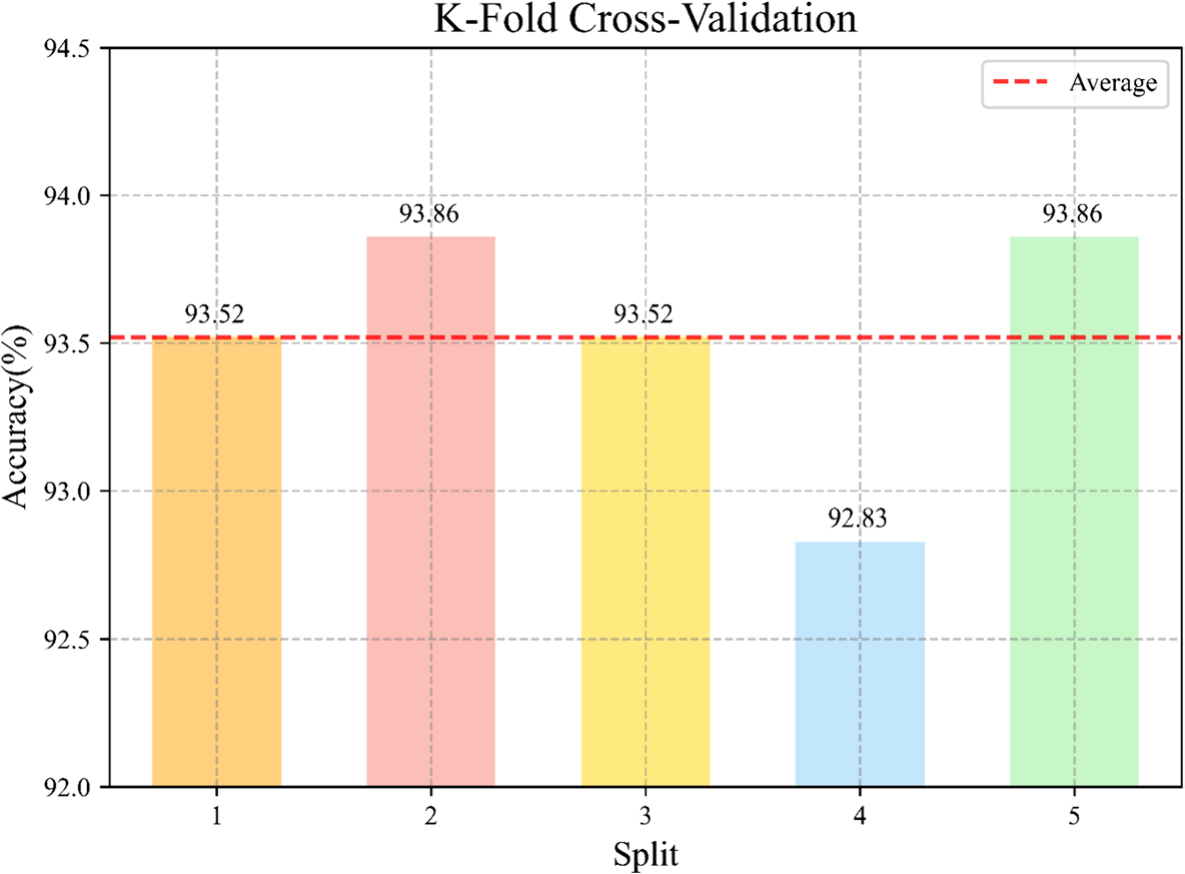
K-Fold cross-validation. The horizontal axis is five different test set splits, and the vertical axis is the accuracy

The evaluation values of our proposed VGG16-CBAM model and other CNNs on prediction indicated that our proposed model achieved better performance in our *Rhinolophus* classification task (Table 2). The F1 score of the VGG16-CBAM was 93.09%, the highest among all tested models. Compared with the VGG16 model, our proposed model VGG16-CBAM model achieved a 1.02% improvement in accuracy, while significantly reducing the number of parameters and the computational effort. It maintained only 22.7% of the original number of parameters and 66.0% of the original FLOPs. VGG16-CBAM for each species also had a more than 86% F1 score in the classification (Table 3). Moreover, we split the data into five different test sets as non-repetitively as possible, and tested each data set separately, averaging the results obtained was 93.52%, and the deviation between the results obtained for each test set and the average value did not exceed $\pm$ 0.69% (Fig. 4). By incorporating cross-validation, we further accurately validate our model.

**Table 2:** Comparison of different networks on 7 *Rhinolophus* species classifications. Params denote the number of parameters of the model; FLOPs is understood as the amount of computation and can be used to measure the complexity of the model, and the unit of throughput is images. F1 score is the ratio of the product of twice the precision and recall to the sum of precision and recall

	Params	FLOPs	Accuracy	F1 Score
AlexNet	61.10 M	715.54 M	82.26%	82.54%
ViT-B/16	86.56M	17.56G	83.28%	83.82%
ResNet50	25.56 M	4.12G	89.08%	89.52%
MobileNetV2	**3.50 M**	**320.24 M**	90.78%	90.92%
VGG16	138.37 M	31.01G	91.13%	91.17%
**VGG16-CBAM**	31.35 M	20.47G	**92.15%**	**93.09%**

**Table 3:** Precision, recall and F1 score of the VGG16-CBAM model for each species. The F1 score here refers to the macro F1 score, which is calculated for each category first and then averaged

Species	Precision	Recall	F1 score
*Rhinolophus affinis*	90.00%	90.00%	90.00%
*R. macrotis*	93.44%	96.61%	95.00%
*R. nippon*	100%	93.33%	96.55%
*R. pearsonii*	93.02%	95.24%	94.12%
*R. pernigei*	100%	100%	100%
*R. pusillus*	89.66%	83.87%	86.67%
*R. sinicus*	86.72%	92.00%	89.32%

**Fig. 5:**
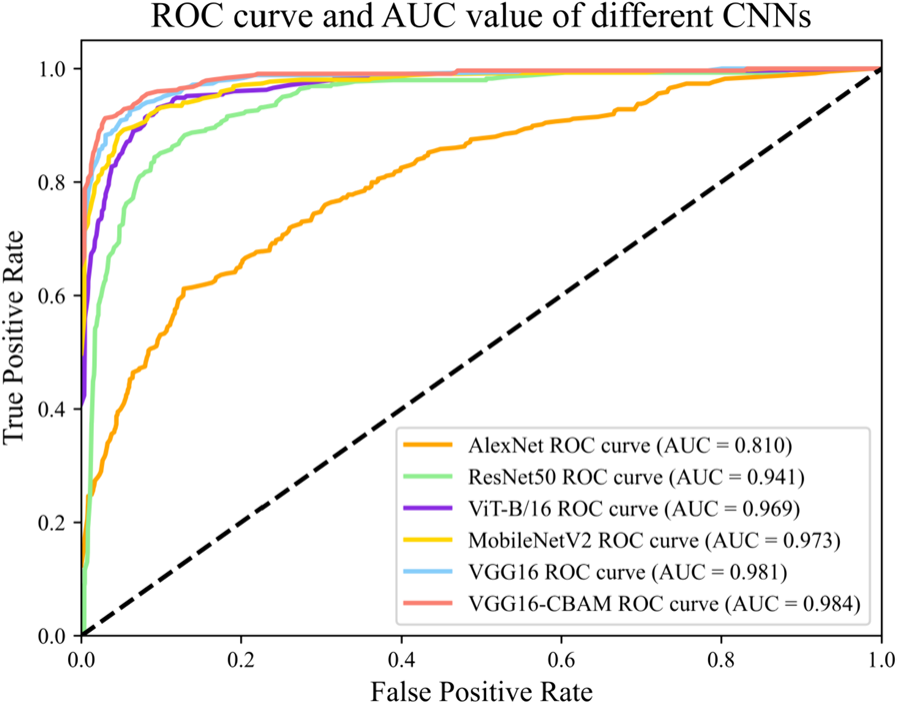
Receiver operating characteristic (ROC) curve and area under the curve (AUC) values of different CNNs. The horizontal axis is the false positive rate and the vertical axis is the true positive

**Fig. 6:**
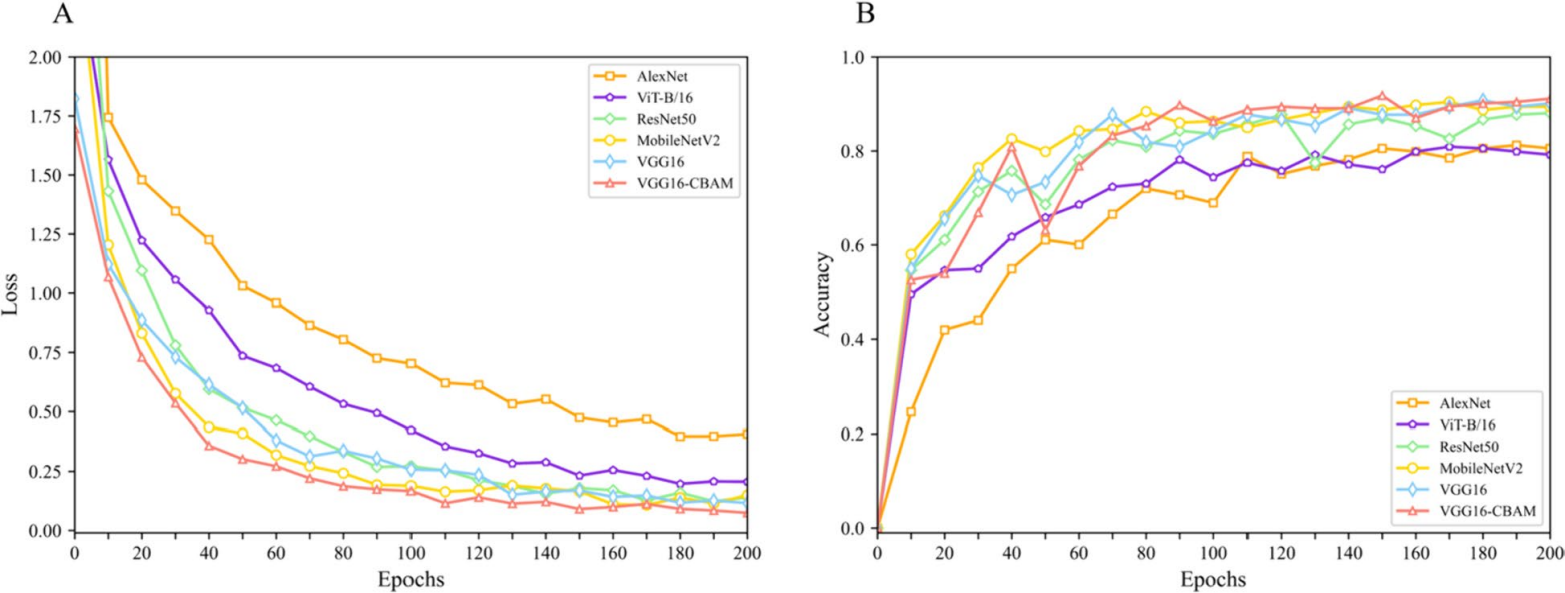
The loss function (**A**) and test accuracy (**B**) for six models

Among the ROC curves and calculated the AUC values of six different CNN models, VGG16 and VGG16-CBAM had better performance, with VGG16-CBAM achieving an AUC of 0.984 and VGG16 achieving an AUC of 0.981 (Fig. 5). These results further demonstrated the good performance of our proposed model for *Rhinolophus* classification. The VGG16-CBAM model had the lowest training loss and highest validation accuracy compared to the other models (Fig. 6). This suggested that the VGG16-CBAM model had the best ability to fit the training data and generalize to new data. Furthermore, the proximity of the training and validation curves of the VGG16-CBAM model suggested that the model did not overfit the training data. Overall, the training and validation curves provided additional evidence that the VGG16-CBAM model outperformed the other models in terms of both training and validation metrics (Fig. 6).

### Visualization of discriminative regions

**Fig. 7:**
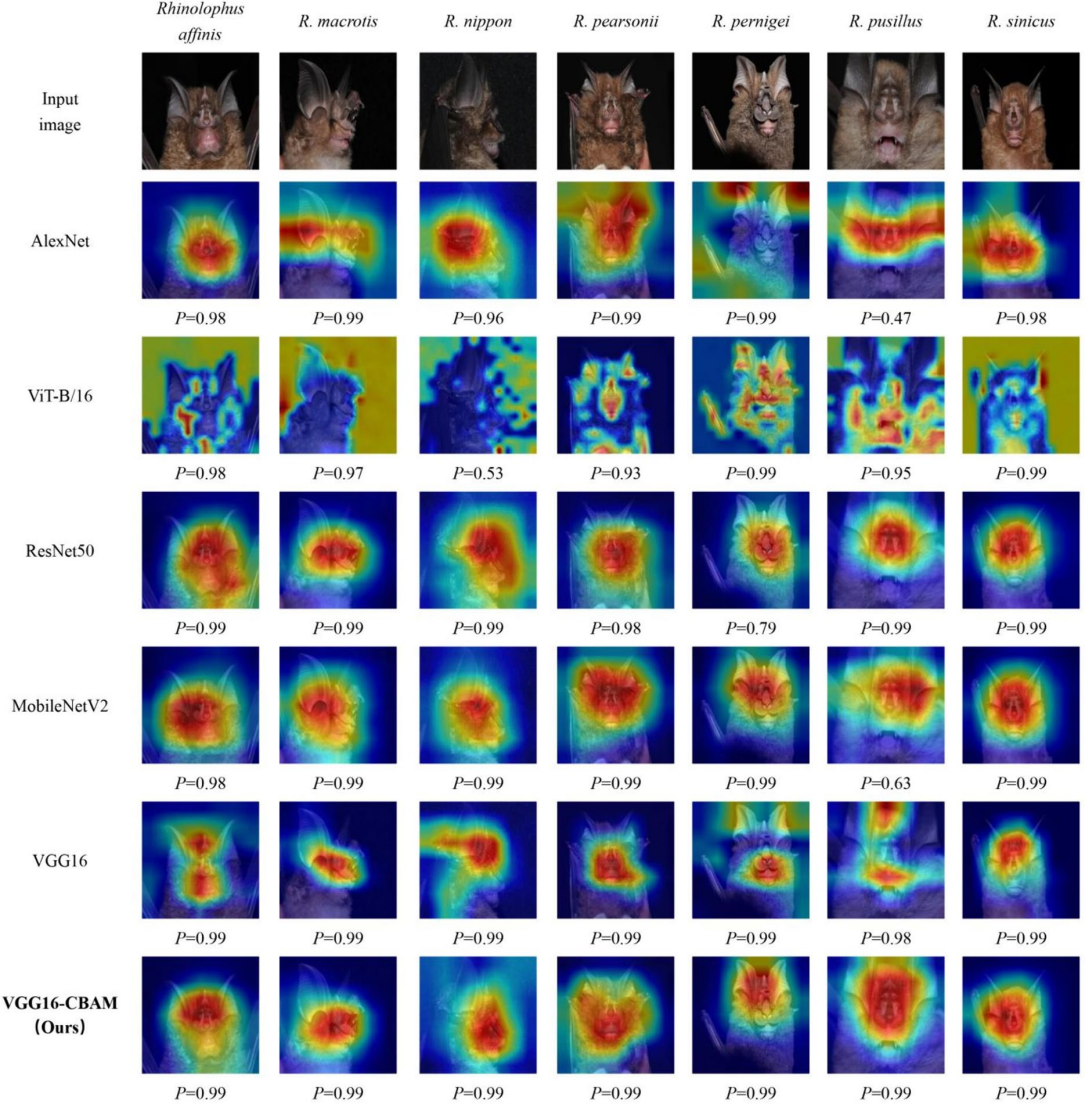
Grad-CAM visualization results. The ground-truth label is shown on the top of each input image, and *P* denotes the Softmax score of each network for the ground-truth class. Note that VGG16-CBAM model locate and identify the discriminative objects and parts of bats, including ear and noseleaf structures. *P* values of all models in different species are more than 0.9, except for some species in AlexNet, MobileNetV2 and ViT-B/16

The Grad-CAM visualization method is a valuable tool to analyze the performance of different network architectures. It uses gradients to produce a localization map highlighting the important regions in the image, which enables the determination of the features used by the network for recognition and classification. Our VGG16-CBAM model could locate and identify the discriminative objects and parts of bats, including ear and noseleaf structures, which could contribute to its superior classification accuracy (Fig. 7 and Table 4). The *P* values of all models in different species are more than 0.9, except for AlexNet (*P* = 0.47) and MobileNetV2 (*P* = 0.63) models in *R. pusillus* and ViT-B/16 (*P* = 0.53) in *R. nippon* (Fig. 7).

**Table 4:** Key for the 7 common horseshoe bats from South China. Wt represents body mass (g), FA represents the length of forearm (mm), Ear represents the length of ear (mm)

Species	Wt	FA	Ear	Horseshoe	Sella	Connecting process	Lancet
*Rhinolophus affinis*	9–19	46–56	15–21	relatively broad but not cover whole muzzle, with well-defined emargination	slightly concave and pandurate in shape	rounded and sparsely haired	parallel-sided lancet
*Rhinolophus macrotis*	4.4–9.5	39–48	18–27	broad, covers muzzle	long and upward pointed, broad, rounded, roughly tongue-shaped, and covered in long	high and rounded, almost parallel to sella at lower part	long, with convex or straight lateral margins and rounded or a little pointed tip
*Rhinolophus nippon*	13–44	53–64	18–29	narrow, not cover muzzle	naked, relatively small, and curved forward	rounded and much higher than tip of sella	hastate or subtriangular lancet
*Rhinolophus pearsonii*	13–20	47–56	23–29	wide, completely cover the muzzle	widened basally, suddenly constricted for the rest, being parallel-sided up to the rounded tip	originates at the sella tip and forms a low round arch	nearly parallel-sided
*Rhinolophus pernigei*	27–37	58–81	28–44	wide and projects in front and on either side beyond the upper lip, with a distinct and very deep median emargination that separates the horseshoe in two sides	parallel-sided, and deflected downward and forward at the tip, with large circular lappets at the base	broadly rounded and heavily reduced	a well-developed and subacutely pointed lancet
*Rhinolophus pusillus*	3.3–8	33–40	33–40	relatively wide with small median emargination	slightly constricted medially, gradually narrowing to widely rounded tip	generally triangular	variable lancet that ranges from elongate with concave sides to short and nearly triangular with parallel sides
*Rhinolophus sinicus*	8.9–10.9	43–56	15–20	relatively wide but not completely cover muzzle, and with visible and well-developed lateral leaflets	virtually parallel sided, and widely rounded off at tip	rounded	hastate lancet that constricts before variably long to short tip

We observe that unlike traditional CNNs, which concentrate on patches of feature regions, ViT-B/16 with transformer architecture emphasizes more dispersed feature regions. Meanwhile the higher error rates of AlexNet (17.74%) and ViT-B/16 (16.72%), in both cases focus on the background area and ignore the features of the bats themselves (Fig. 7). For the better performing models, the region of interest was mainly on the face of the bat, but we note that the regions of interest of these models cross but are not identical, for example, on the *R. nippon* image, the region of interest of MobileNetV2 contains the ear and the face, VGG16 focuses on the face and some body parts, while VGG16-CBAM mainly focuses on the noseleaf and face (Fig. 7). Meanwhile, we note that VGG16 clearly shows that the model mainly focuses on the noseleaf and mouth, but also focuses more on the ear when judging the image shown in *R. pernigei*. In addition, MobileNetV2 focuses on a larger feature area (more features of interest), while VGG16-CBAM and VGG16 focus on more concentrated areas compared to the former (Fig. 7).

## Discussion

**Fig. 8:**
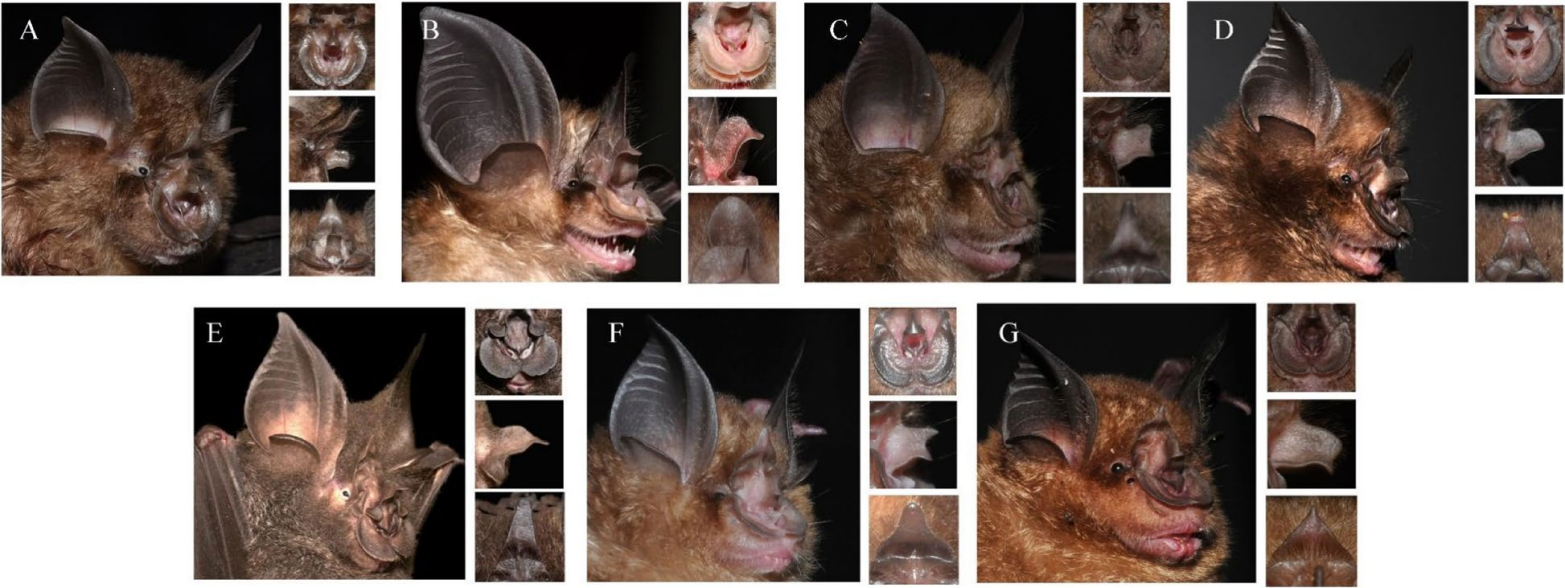
Distinctive head characteristics of 7 common horseshoe bats from South China. Head features of each species are displayed: **A** *Rhinolophus affinis,* **B** *R. macrotis,* **C** *R. nippon,* **D** *R. pearsonii,* **E** *R. pernigei,* **F** *R. pusillus,* and **G** *R. sinicus*. The right side of the head images, from top to bottom, exhibits: top view of the horseshoe and the sella, lateral view of the connecting process, and side view of the lancet. These aforementioned characteristics are routinely employed in the species identification of horseshoe bats

DCNNs are becoming increasingly important across various scientific disciplines including mammalogy, such as taxonomic identification [36, 37], individual recognition [16, 38, 39], and monitoring [40]. While VGG16 is a classical CNN that uses a smaller convolution kernel to increase the network depth and obtain a larger field of vision [41–44], its overwhelmingly large number of parameters requires significant memory and computational resources (Table 2). We also observed a slow convergence of the loss function and a tendency toward overfitting during training (Fig. 6). After introducing CBAM and modifying the fully connected layer of VGG16 by adding a convolutional layer and global average pooling to reduce the number of parameters and improve regularization, VGG16-CBAM achieved the highest classification accuracy of 92.15%, outperforming other popular models such as VGG16, AlexNet, MobileNetV2, and ResNet50 (Fig. 5 and Table 2). In our model, *R. pernigei* can achieve 100% accuracy, suggesting that this species is easy to identify, and distinct from other 6 species. Considering taxonomic identification, this species, owing to its superior body size, is undoubtedly the most distinguishable among the seven *Rhinolophus* species, attributable to its highly specialized noseleaf lobes and comparatively larger ear (Table 4 and Fig. 8) [1]. But, our confusion matrix of VGG16-CBAM indicated misclassification within cases in some species, e.g., *R. affinis* (10%), *R. pusillus* (16%), and *R. pearsonii* (5%). Our model confused *R. affinis* with *R. sinicus* and *R. pusillus* due to the procession of the broad horseshoe with well-defined emargination and lost details caused by the resolution reduction protocol. Notably, these misclassified species could also be distinguished by their external measurements such as the length of the forearm or length of the ear (Table 4 and Fig. 8). We believe the utilization of various data and evidence (e.g., morphological measurements, good-quality images covering potential key regions, echolocation signals, etc.) could improve the accuracy of species recognition.

The training data are the foundation of the deep learning task in species recognition [32]. A larger training data set can provide a more comprehensive representation of the species, including various characteristics, environments, and lighting conditions, which substantially enhance the model’s ability to learn and prevent the model from overfitting [45]. Therefore, the emphasis on training dataset size is not only a matter of quantity but also a strategic approach to improve model generalization and reliability. In our study, we also recognized the importance of dataset size in training. The 879 images of 7 *Rhinolophus* bat species, as listed in Tables 1 and 4, represent one of the largest known image datasets thus far for fine-grained classification of bats. However, it remains insufficient for the demands of CNNs. To counterbalance this limitation, we strategically employed a range of data augmentation techniques, such as grid-like masking over images, random noise and blurring, rotation images, and color transformation [26, 46]. After enriching our training dataset to add 694 images, all models performed with high accuracy from 82.26% to 92.15%, suggesting that the integration of these methods to enrich the training dataset could improve the model’s accuracy and generalization ability. Our study also highlights the importance of employing various methods to process images during model training, providing a range of possibilities to train the best model [47–52].

One method to comprehend what is being learned by the DCNNs is to visualize the feature heat maps [53, 54]. Grad-CAM visualization could provide insights into the model’s decision-making process and highlight the areas of an image that are most influential in determining the predicted class [55]. The high SoftMax score was potentially achieved because we have fewer categories and our background was relatively homogeneous, which had less influence on the network classification. The success of this recognition and classification task relied on learning the correct features with discriminative power, and our model achieved this. Nevertheless, the Grad-CAM method is not without its constraints. It adeptly discerns superior from inferior models, yet struggles to the proficient models. Despite these limitations, it is irrefutable that in certain cases, the model's visualization has sparked innovative ideas for novel classification methodologies. This result indicates that the application of DCNNs could facilitate new avenues for the development and optimization of future classification systems. Meanwhile, it was observed that that DCNN models appeared to capture specific individual traits, such as fur color, the characteristics, and relative sizes of horseshoe, nose, and ear. These features are less employed in classification of horseshoe bat (Figs. 6 and 8, Table 4). The distinctions observed in these assessed characteristics could stem from variations between two-dimensional images and actual three-dimensional specimens. Additionally, these discrepancies may not necessarily indicate genuine differences between species, but rather differences within the sampled populations. These are worthy of our in-depth study in both data sets and models.

Finally, we proposed that image recognition in deep learning is a promising yet rapidly evolving research field with varying effectiveness and performance across different models and architectures [56]. To achieve accurate taxonomic classification using image recognition algorithms, machine learning experts and taxonomists need to cooperate intimately and explore the compatible models tailored to their specific circumstances [24]. Another concern regarding simple application scenarios involves creating a lightweight, efficient, and portable app or software [57]. In the era of artificial intelligence and big data, taxonomists need to embrace this state-to-art technology to facilitate species discrimination and optimize taxonomic settings such as comparing focusing regions by human and model analyses. Groups without distinct interspecific variations (e.g., rodents, shrews, and bats) may be challenging for the model. Subsequently, incorporations of taxonomic systems/keys and optimization of the data sources, types, and architectures of models are proposed. This includes building a standardized photography protocol, emphasizing concerning areas, minimizing background influence, or even incorporating sources covering more taxonomical signals (e.g., skulls, teeth, pelage or other external features). The development of species identification models capable of integrating multiple data types such as metric data, morphological descriptions, images, and sounds are recommended.

## Conclusions

In this study, we demonstrated the effectiveness of deep convolutional neural networks for classifying 7 horseshoe bat taxa with high inter-species similarity and intra-species variations from South China. Based on 879 images collected from 9 years of field surveys, our model of VGG16-CBAM has the highest accuracy (92.15%) compared with AlexNet, MobileNetV2, ResNet50, ViT-B/16, and VGG16. We also analyzed how deep learning models achieved this high classification accuracy by localizing hot discriminative regions. Our results indicated that deep learning models learned similar discriminators from the noseleaf and ear of horseshoe bats, which are commonly used by human experts. In the epoch of artificial intelligence, we hope our findings will inspire further research on image-based automatic classification of species and potentially provide substantial advantages in addressing the crux within biology—identification of taxon—through the application of innovative methodologies and data.

## Data Availability

All the original images of species, the related testing models, and source codes for this work have been released publicly at Zenodo. https://zenodo.org/records/10613387.
